# Seed-Applied Cobalt, Molybdenum, and Nickel Improve Nitrogen Metabolism in Soybean Plants Across Seed Vigor Levels

**DOI:** 10.3390/plants14213368

**Published:** 2025-11-04

**Authors:** Abimael dos Santos Carmo-Filho, Carlos Henrique Queiroz Rego, Glória de Freitas Rocha Ribeiro, Rafael Mateus Alves, Lucas Alves de Almeida, Bruna Wurr Rodak, José Lavres, Francisco Guilhien Gomes-Junior

**Affiliations:** 1 Department of Crop Science, Luiz de Queiroz College of Agriculture, University of Sao Paulo, Piracicaba 13418-900, SP, Brazil; carlosqueirozagro@gmail.com (C.H.Q.R.); glorinharribeiro@gmail.com (G.d.F.R.R.); rafaelmateusalves@usp.br (R.M.A.); francisco1@usp.br (F.G.G.-J.); 2 Center for Nuclear Energy in Agriculture, University of Sao Paulo, Piracicaba 13418-900, SP, Brazil; lucas_alvesdealmeida@usp.br (L.A.d.A.); bruwurr@gmail.com (B.W.R.); jlavres@cena.usp.br (J.L.)

**Keywords:** *Glycine max* (L.) Merril, seed inoculation, nitrogen assimilation, micronutrients, physiological potential of seeds

## Abstract

Cobalt, molybdenum, and nickel are elements directly involved in biological nitrogen fixation in legume plants. However, there is a lack of information about the effects of the interaction among these elements on seed vigor and plant development. This study aimed to evaluate the effects of different doses of these elements on soybean seeds with higher and lower vigor, focusing on nitrogen metabolism and plant development under controlled conditions. The two lots of soybean seeds (higher and lower vigor) were treated with doses of 0, 2, 4, 6, and 8 mL kg^−1^ of seeds of a liquid commercial product composed of cobalt, molybdenum, and nickel. At the full flowering stage, urease and nitrogenase activities, dry biomass of shoots, roots, and nodules, nitrogen concentration in shoots, plant height, number of nodules, and the efficiency of biological nitrogen fixation (measured by nitrogen-15 isotopic ratio) were assessed. Urease activity increased by 191% in high-vigor seed plants and 65% in low-vigor seed plants. Nitrogenase activity was higher in higher-vigor plants. Nodule dry biomass increased by 42% in lower-vigor plants compared to the control treatment, while in higher-vigor plants, it decreased with increasing doses. Shoot biomass was 30% higher than the control at the 2 mL kg^−1^ dose. In general, the best responses to the application of the elements in the evaluated variables were observed with the doses of 2 and 4 mL kg^−1^. It is concluded that the appropriate application of cobalt, molybdenum, and nickel on seeds enhances growth and symbiotic efficiency. However, excessive doses may cause phytotoxic effects.

## 1. Introduction

Soybean (*Glycine max* (L.) Merril) is a globally cultivated crop for protein and oil and ranks among the most important crops worldwide, mainly used for the production of human and animal food [1,2]. According to a survey of the foreign agricultural service of the US Department of Agriculture [3], Brazil accounts for 39% of the 400 million tons produced worldwide. For the 2024/2025 crop season, it is estimated that the soybean production is 169,605.8 thousand tons in 47,619.8 thousand hectares cultivated in Brazil [4].

By presenting high protein levels, soybeans have a high nitrogen (N) requirement, which is mostly met through biological nitrogen fixation (BNF) [5]. This process occurs through a symbiotic association between legumes and bacteria, commonly known as rhizobia, which infect root hairs. As a result of this association, specialized structures called nodules are formed. In these specialized organs, the bacteria convert atmospheric nitrogen (N_2_) into forms usable by the plant, while the plant provides photosynthetic products to sustain bacterial metabolism [6,7,8].

Cobalt (Co), molybdenum (Mo), and nickel (Ni) elements are fundamental to the proper functioning of BNF and, consequently, for N metabolism and plant development. Co is recognized as a beneficial element for plants [9]. In legumes such as soybean, it plays a key role in N metabolism, acting synergistically in the processes of nodulation and BNF [10]. It is an essential component of cobalamin, indispensable for the activity of various enzymes and coenzymes [11], and is involved in the formation of leghemoglobin [12], a protein responsible for regulating O_2_ to optimal levels within the nodules [13].

Molybdenum is a micronutrient required for plant growth and development, playing a crucial role in the biosynthesis of molybdate-dependent enzymes. These enzymes are involved in key biological processes, such as nitrate assimilation, phytohormone biosynthesis, purine metabolism, and sulfite detoxification. In the context of N metabolism, Mo acts as a cofactor for the nitrate reductase enzyme, which is critical for the efficiency of nitrogen assimilation by plants [14,15,16] and for the nitrogenase enzyme [17], which reduces N_2_ to ammonia (NH_3_) during BNF [18].

Ni is also an essential element for plants [19]. This micronutrient serves as a cofactor for two metalloenzymes, urease [20] and hydrogenase [21], which are directly involved in N metabolism [22]. Urease catalyzes the conversion of urea into ammonium ions (NH_4_^+^), enhancing the efficiency of N uptake. In the absence of Ni, its activity is nonexistent, resulting in the accumulation of unmetabolized urea at toxic levels in plant tissues [23]. Meanwhile, the hydrogenase enzyme, during BNF, reuses the H_2_ produced in biological nitrogen fixation, reducing energy loss in nitrogenase catalysis [24].

In addition to the BNF, the proper establishment of seedlings at the beginning of their cultivation is critical to obtaining high yields in the soybean crop. In this context, the seed vigor plays a central role, as it reflects the capacity of the seeds to promote uniform and vigorous seedling development under adverse environmental conditions [25,26]. As a strategy to assist the performance of seedlings, the treatment of seeds with a mixture of mineral elements has often been adopted [27,28,29]. However, depending on the dose used, it can cause phytotoxicity, impairing germination and development of seedlings [30,31,32].

Although the benefits of isolated Co, Mo, and Ni supply are well-documented for the BNF, there is a shortage of information in the literature on the effects of the joint application of these elements via seed treatment, considering seeds with different levels of vigor. It is unclear whether the vigor of seeds influences the efficiency of absorption and use of Co, Mo, and Ni, nor how this interaction affects nitrogen metabolism and the development of soybean plants.

Given this gap, this study investigates how Co, Mo, and Ni influence N metabolism in soybean across seed vigor levels.

## 2. Results

### 2.1. N Metabolism

**Figure 1:**
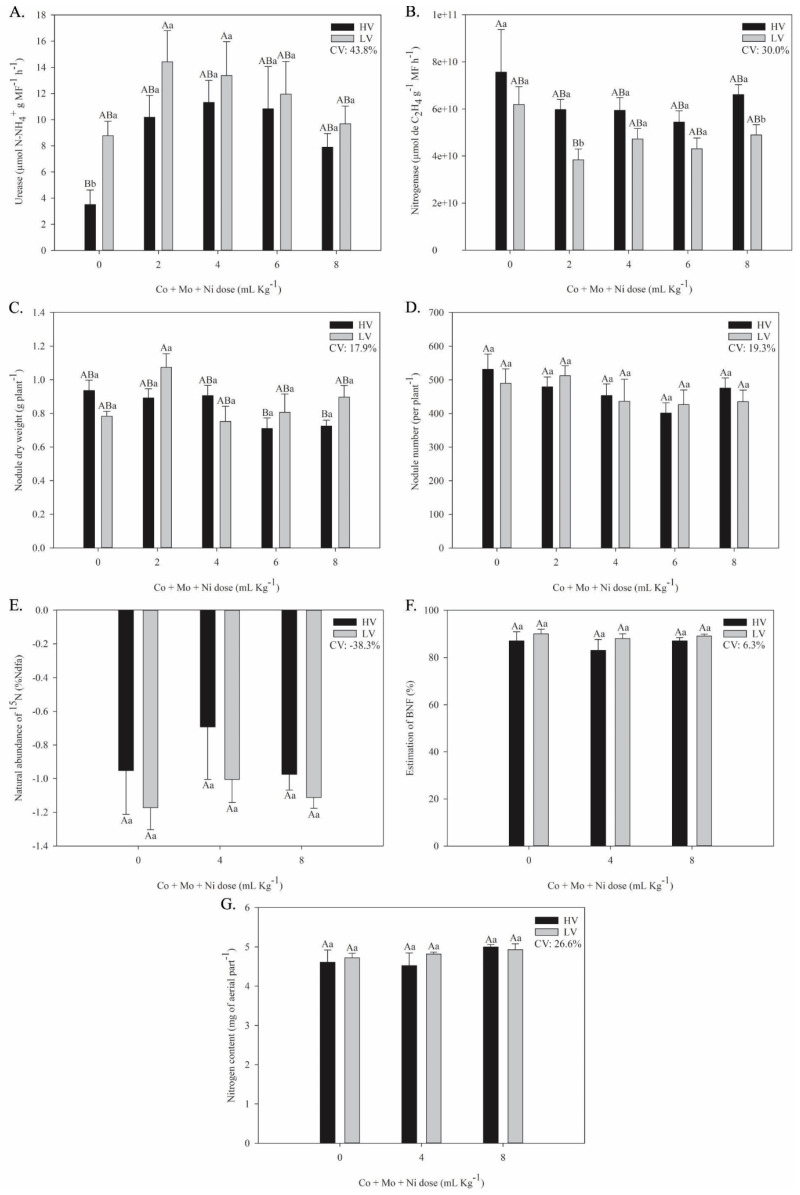
N metabolism in soybean plants evaluated at the R2 stage, from seeds with higher vigor (HV) and lower vigor (LV) treated with doses of Co + Mo + Ni. Urease activity (**A**), nitrogenase activity (**B**), nodule dry weight (**C**), nodule number (**D**), natural abundance of δ^15^N‰ (%Ndfa Rice: 4.94) (**E**), BNF estimation (**F**), and nitrogen concentration (**G**). Means with identical capital letters do not differ between treatments in all columns, while identical lowercase letters do not differ between treatments within each dose by the Tukey test (*p* ≤ 0.05). Mean accompanied by standard error; n = 5.

The application of mineral element doses positively influenced certain response variables related to N metabolism (Figure 1). Urease activity increased following treatment, with values rising by 191% in plants from high-vigor (HV) seeds and by 65% in plants from low-vigor (LV) seeds relative to the control. A significant difference between seed lots was observed only in the control treatment, where plants from LV seeds exhibited higher enzyme activity. These results suggest that the mineral treatments enhanced urease activity consistently across seed lots, regardless of their vigor (Figure 1A).

Regarding nitrogenase activity, a significant reduction of 49% was observed between the control treatment (0 mL kg^−1^) of HV and the 2 mL kg^−1^ dose of LV plants. Concerning the effect of seed vigor, HV plants exhibited higher nitrogenase activity than LV plants, with statistically significant differences at the 2 mL kg^−1^ and 8 mL kg^−1^ application rates (Figure 1B).

In LV plants, applying 2 mL kg^−1^ of Co, Mo, and Ni increased nodule dry mass by 42% relative to the control from the same seed lot. However, higher doses (6 and 8 mL kg^−1^) significantly reduced nodule dry mass in HV plants compared to LV plants (Figure 1C). Conversely, no significant differences were observed in the number of nodules with increasing doses or due to the interaction between seed vigor and dose across both seed lots (Figure 1D).

Regarding the percentage of nitrogen in the plant derived from the atmosphere (%Ndfa), neither seed vigor nor the application of Co + Mo + Ni doses had a significant effect (Figure 1E). Across treatments, approximately 85–88% of the N in the plants originated from atmospheric sources, indicating effective BNF (Figure 1F). Similarly, no significant differences were observed in the total nitrogen content of the shoot among the treatments with Co + Mo + Ni application (Figure 1G). With the application of 4 and 8 mL kg^−1^ doses of Co + Mo + Ni, δ^15^N values of −0.8‰ and −1.0‰, respectively, were observed. In contrast, rice plants (non-N_2_-fixers) grown under the same climatic and substrate conditions exhibited an average δ^15^N value of +4.94‰.

### 2.2. Plant Development

**Figure 2:**
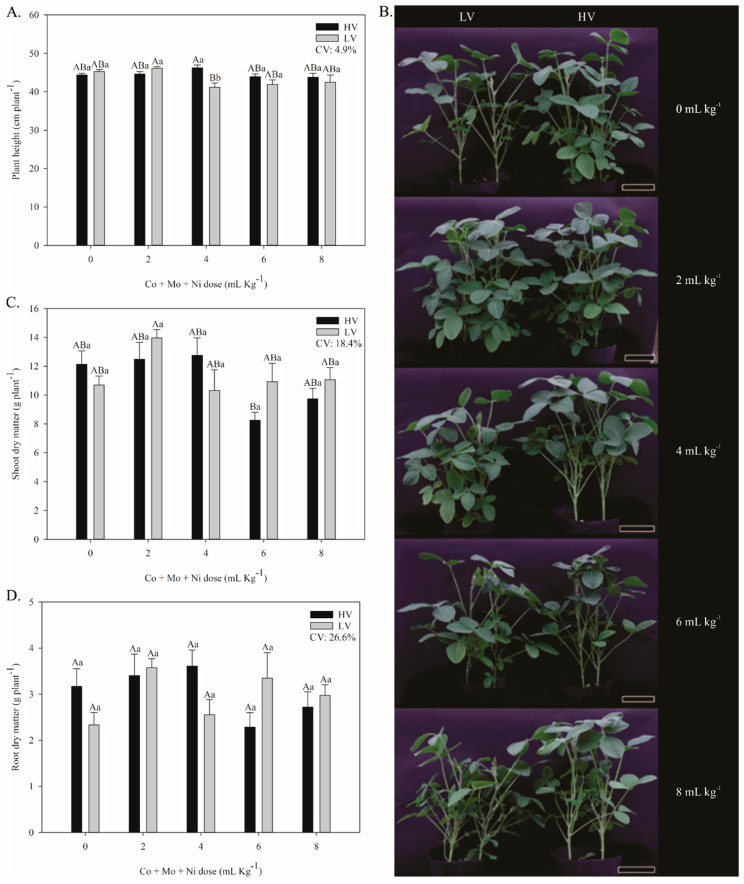
Development analysis of soybean plants evaluated at the R2 stage, from seeds with higher vigor (HV) and lower vigor (LV) treated with doses of Co + Mo + Ni. Height (**A**), visual illustration of growth (Bars: 11.5 cm) (**B**), shoot dry matter (**C**), and root dry matter (**D**). Means with identical capital letters do not differ between treatments in all columns, while identical lowercase letters do not differ between treatments within each dose by the Tukey test (*p* ≤ 0.05). Mean accompanied by standard error; n = 5.

Plant height was influenced by the application of Co + Mo + Ni. Our results indicate that a dose of 4 mL kg^−1^ significantly reduced the height of low-vigor (LV) plants by approximately 11% relative to those treated with 2 mL kg^−1^ from the same seed lot. From 6 mL kg^−1^ onward, a reduction in plant height was observed in both seed lots (Figure 2A,B).

Regarding shoot dry mass, the application of 2 mL kg^−1^ resulted in a 30% increase in LV plants relative to the control. However, starting at 4 mL kg^−1^, a reduction in shoot dry mass was observed in plants from both seed lots (Figure 2C). A similar trend was noted for root dry mass, which also decreased with increasing doses of Co + Mo + Ni (Figure 2D).

## 3. Discussion

The findings of this study provide compelling evidence for the agronomic and physiological value of seed treatments combining Co, Mo, and Ni in soybean cultivation.

We observed an increase in urease enzyme activity relative to the control treatment. This occurred due to the contribution of the element Ni, since this micronutrient is a cofactor of the urease enzyme [33], which catalyzes the hydrolysis of urea into NH_3_, CO_2_, and water, thus being fundamental in the use of N by plants [34]. Other studies also reported increased urease activity in soybean following Ni seed treatment compared to untreated controls [35,36,37]. Although the supply of Ni increased urease enzyme activity compared to the control, the elevation of doses (6 and 8 mL kg^−1^) resulted in a reduction in the measurable accumulation of this enzyme, suggesting a phytotoxic effect at higher levels of the micronutrient. This behavior indicates that Ni, although essential as a urease cofactor, can impair N metabolism when applied in excess. Similar results were reported by Khan et al. [38], who observed an initial increase in urease activity followed by a decrease with the elevation of Ni concentrations in soybean plants. On the other hand, low urease activity can lead to the accumulation of urea in plant leaves and, ultimately, compromise N metabolism [39]. The use of Ni in adequate doses is, therefore, essential to allow better use of N by soybean plants by increasing the activity of urease.

The activity of the nitrogenase enzyme from the technique of reducing acetylene to ethylene is widely used to indirectly observe the efficiency of BNF, since nitrogenase also reduces N_2_ inside the nodules during BNF [40]. The greater nitrogenase activity observed for the HV plants is probably related to the rapid emergence of seedlings, which may have enabled better development and establishment of the nodules, at the beginning of vegetative development, through symbiosis with rhizobia, in relation to plants originating from LV. According to Ebone et al. [41], plants from seeds with high vigor have better development conditions due to rapid emergence at the beginning of establishment, which results in faster metabolic performance compared to plants with late emergence, that is, those obtained from seeds with lower vigor. However, it is important to emphasize that seed vigor has a considerable influence only on crop establishment; from then on, plant development will be governed by the environment [42].

The lack of influence of doses and vigor level on the number of nodules is probably due to the establishment of the number of nodules being defined by the density of symbiotic microorganisms, imposed by the inoculation of the product containing the bacteria since the same dose was used for all treatments, because according to Moretti et al. [43], most nodules are formed through the addition of the inoculant to the roots and there is an increase in nodulation as the number of inoculations increases. In addition, it is important to emphasize that nodule formation is regulated by the plant’s N demand. Thus, in the presence of available N in the substrate or under conditions of high N accumulation in plant tissues, soybean may activate an autoregulatory mechanism of nodulation, leading to the suppression of nodule formation [44].

The total N accumulation in soybean plants from seeds treated with 4 and 8 mL kg^−1^ of Co + Mo + Ni (81 and 78.5 mg N per aerial part, respectively) was higher than in plants from the control treatment (70 mg N per aerial part), and markedly greater than the value observed in rice plants (16 mg N per aerial part). This highlights the contribution of these doses to nitrogen metabolism. The accumulation of N in soybean plants through the quantitative contribution of BNF can be interpreted by the difference between the abundance of δ^15^N of N-fixing plants (soybean) and that of non-fixing reference plants (rice), grown in the same location, since plants that do not produce BNF accumulate only the N available in the soil [45].

The reduction observed in plant height may be associated with a phytotoxic effect of the seed treatment, mainly due to the elements Co and Ni. High concentrations of Co can cause symptoms of phytotoxicity in plants by decreasing iron absorption and, consequently, causing stunting [46]. Levy et al. [47] observed that excessive application of Ni can also reduce the height of soybean plants due to its phytotoxic potential. In the literature, there are no reports of toxicity in soybean plants caused by Mo. The occurrence of phytotoxicity in plants by this micronutrient is very rare and is usually subject to diagnostic errors, due to symptoms linked to the altered activities of many redox enzymes [48].

Similarly, the increased doses probably caused phytotoxicity in the plants of the HV lot, leading to a decrease in the dry mass of the aerial part and root. Acha et al. [49] and Lavres et al. [35] also observed a reduction in the dry mass of the aerial part of soybean plants after application of high doses of mineral elements in the seed treatment. According to Sreekanth et al. [50], when Ni is supplied in high doses, it can interfere with the development, photosynthesis, mineral nutrition, and enzymatic activity of the plants, which subsequently reflects in low dry mass accumulation. Excess Co can also cause a decrease in dry mass accumulation, due to the occurrence of oxidative stress, which affects nitrogen metabolism, photosynthesis, and the antioxidant system [51].

The discrepancy observed in the values obtained for the dry mass of the aerial part and root between the lots reinforces, again, that seed vigor did not interfere with the effects of the doses on plant development. Mondo et al. [52] stated that seed vigor is directly associated with the initial growth of the plants; therefore, its effects do not last throughout the crop cycle. However, the use of soybean seeds with high vigor is essential for establishing an adequate plant population density, indirectly impacting grain productivity [53].

In general, elements such as Co, Mo, and Ni are normally required by plants in smaller quantities. Hence, when applied in high doses, they can be detrimental to their performance. However, when seed treatment is carried out with an adequate supply of these elements, it can reduce nutrient losses and consequently aid in plant development, especially in the initial stages of crop establishment [54]. Furthermore, by enhancing key processes of N-metabolism—particularly urease and nitrogenase activities—and improving early plant development, such treatments may represent an efficient strategy to enhance BNF and crop productivity, particularly when seed vigor is suboptimal. It is important to highlight that different combinations and concentrations of Co, Mo, and Ni may exert synergistic or antagonistic effects on the evaluated parameters. Therefore, further research is needed to elucidate these interactions and their potential impacts on nitrogen metabolism.

## 4. Materials and Methods

The experiment was carried out at the Department of Crop Science of the ‘Luiz de Queiroz’ College of Agriculture, University of São Paulo (ESALQ/USP) and the analyses were performed at the Seed Analysis Laboratory and Image Analysis Laboratory of ESALQ/USP and at the Stable Isotope Laboratory of the Center for Nuclear Energy in Agriculture of the University of São Paulo (CENA/USP), in the city of Piracicaba, São Paulo, Brazil.

### 4.1. Characterization of the Seed Lots

Initially, two lots of soybean seeds of the M5917 IPRO cultivar of indeterminate growth and relative maturity group 5.9 were characterized with tests to evaluate the physiological potential: germination (GER), first germination count (FGC) [55], accelerated aging with saturated sodium chloride (NaCl) solution (AASS) and electrical conductivity (EC) [56]; and water content after EASS (WC-AASS) [57]. Based on the results of the physiological potential of the seeds, the lots were characterized as having higher and lower vigor (HV and LV) (Appendix A).

### 4.2. Seed Treatment

Seeds from both HV and LV lots were immediately treated with five doses of a liquid commercial product composed of 22% Mo, 1.89% Co, and 1.0% Ni. The doses evaluated were 0, 2, 4, 6, and 8 mL of the product kg^−1^ of seed. For this purpose, 500 g of seeds from each lot were placed in plastic bags with subsequent application of the treatments and homogenized by shaking for approximately 2 min. Subsequently, the seeds were dried in the laboratory at room temperature.

### 4.3. Experiment Conduction

In a greenhouse, soybean seeds treated with doses of Mo, Co, and Ni were sown in pots (8.5 L capacity) with substrate. The experimental unit consisted of two plants per pot, and the experimental design used was completely randomized, with a 2 (seed vigor levels) × 5 (doses of Co, Mo, and Ni) factorial scheme, with five replicates, totaling 50 pots. During the experiment, the mean temperature and relative humidity of the air were 24 °C and 73%, respectively. The climatic data were measured with a digital thermo-hygrometer.

The substrate used in the experiment was composed of a 3:1:1 ratio of soil (typical Eutroferric Red Nitosol) [58], organic substrate (consisting of pine bark, peat and charcoal) and vermiculite, respectively, resulting in the following chemical characteristics: pH (CaCl_2_) 4.4, organic matter (total soil organic carbon content/Walkey–Black method [59]) 7 g dm^−3^; P (resin) 4 mg dm^−3^, K 0.5 mmolc dm^−3^, Ca 15 mmolc dm^−3^, Mg 5 mmolc dm^−3^ determined by extraction with ion exchange resin [59,60]; H + Al (SMP buffer solution [61]) 28 mmolc dm^−3^; Al (1 N KCl [59]) 3 mmolc dm^−3^; base sum of 21 mmolc dm^−3^, cation exchange capacity (CTC) of 49 mmolc dm^−3^, base saturation (V) of 42%, B (DTPA) 0.16 mg dm^−3^, Mn (DTPA) 7.80 mg dm^−3^, Co (USEPA) 0.05 mg dm^−3^, Mo (USEPA) < 0.20 mg dm^−3^, Ni (USEPA) 0.15 mg dm^−3^, Zn (DTPA) 1.10 mg dm^−3^, Cu (DTPA) 0.42 mg dm^−3^. The substrate acidity correction was carried out 90 days before sowing, to allow the pH to be raised to 6–6.5. 0.23 kg of dolomitic limestone was used, previously homogenized with the substrate in a 400-L concrete mixer for five minutes, before filling the pots.

During the experiment, five seeds were sown per pot at a depth of 2 cm, and thinning was performed ten days after seedling emergence, maintaining two plants per pot. One day before sowing, the substrate in the pots was fertilized with doses of 150 mg dm^−3^ K_2_SO_4_, 200 mg dm^−3^ Ca(H_2_PO_4_)_2_·H_2_O, 0.5 mg dm^−3^ boric acid, 1.0 mg dm^−3^ Cu, 5.0 mg dm^−3^ Mn and 5.0 mg dm^−3^ Zn to meet the demand for K, P, B, Cu, Mn and Zn, respectively. At the V5 phenological stage—plants occurred with four fully expanded trifoliates [62]—a new fertilization was performed only for the macronutrient K at a dose of 150 mg dm^−3^ K_2_SO_4_. For N supply, the seeds were inoculated with the N_2_-fixing bacteria *Bradyrhizobium japonicum* (SEMIA 5079 and SEMIA 5080) at a concentration of 5.0 × 10^9^ colony-forming units per mL, adopting a dose of 4 mL per kg of seed. Concomitantly with the implementation of the soybean experiment, rice plants (*Oryza sativa*) (a non-N_2_-fixing plant) were sown in pots of the same size, substrate, and fertilization (without the application of Co + Mo + Ni doses), but with the application of urea to verify the natural enrichment of δ^15^N‰ available in the substrate.

During the management of the experiment, daily irrigations were carried out to maintain the maximum water retention capacity of the substrate at 60%. The soil relative water content (SRWC) was measured by the weight method [63]. The pots were watered and weighed on a daily basis until they reached their corresponding target SRWC to replace the evaporated and transpired water. SRWC was calculated as follows:

$$\mathrm{SRWC}=\frac{\left(W_{\mathrm{soil}}-W_{\mathrm{pot}}-{\mathrm{DW}}_{\mathrm{solo}}\right)}{\left(W_{\mathrm{FC}}-W_{\mathrm{pot}}-{\mathrm{DW}}_{\mathrm{soil}}\right)}*100$$

where the following applies:

W_soil_ is the current soil weight (soil + pot + water), W_pot_ is the weight of the empty pot, DW_soil_ is the dry soil weight, and W_FC_ is the soil weight at field capacity (soil + pot + water).

For phytosanitary control, the insecticide Orthene 750 BR (active ingredient acephate) and the fungicide Nativo (active ingredients trifloxystrobin and tebuconazole) were applied, following the dose recommended by the manufacturer.

The effects of treatments on N metabolism and plant development were evaluated at the full flowering phenological stage (R2), adopting the methodologies described below.

### 4.4. Assessment of N Metabolism

For assessment of N metabolism, analyses were performed on the activity of urease and nitrogenase, dry matter, and number of nodules, N concentration in the shoot of the plants, as well as the percentage of nitrogen derived from the atmosphere (%Ndfa), employing the method of variation in the natural abundance of δ^15^N‰.

To determine urease activity, the methodologies adapted by [64,65] were used. The third fully expanded leaf from the apex of the plants was collected early in the morning and stored in liquid nitrogen for later analysis in the laboratory. Cross-sections of 1 mm thickness were made from the fresh leaves. Then, 0.2 g of the sample was incubated in 8 mL of sodium phosphate-buffered solution with urea, pH 7.4, at 30 °C for 3 h with constant agitation. In a 0.5 mL aliquot of the extract from the incubation, 2.5 mL of solution I (0.1 mol L^−1^ of phenol and 170 µmol L^−1^ of sodium nitroprusside) and 2.5 mL of solution II (0.125 mol L^−1^ of sodium hydroxide, 0.15 mol L^−1^ of sodium phosphate, and 3% sodium hypochlorite) were added. A new incubation was carried out at 37 °C for 35 min. Then, readings were taken in a spectrophotometer at 625 nm to determine the NH_4_^+^ concentration in the samples; for this purpose, an ammonium chloride standard curve was used.

Nitrogenase activity was evaluated by the acetylene reduction methodology according to Boddey et al. [66], which consists of mimicking nitrogen gas with acetylene, resulting in the formation of ethylene. For this purpose, the plant roots were removed from the substrate, and three nodules with an average size of 2.22 mm in diameter were collected and placed in tubes; then the tubes were closed with airtight caps. With the aid of a syringe and needle, 1 mL of air was removed from the tube, approximately 10% of the gas phase of the tubes, then 1 mL of acetylene gas, with a purity of 98–99%, was injected into the tubes. After 15 min of incubation, 1 mL of the gas phase was transferred to the vacuum tubes. Subsequently (approximately 1 h), 1 mL of the sample was injected for ethylene determination in a Thermo Fisher Scientific^®^ gas chromatograph, model Trace 2000GC, with a Porapak N column (1.8 m), flame ionization detector, and constant pressure flow of 120 KPa.

The dry matter and number of nodules were determined in the roots of the two plants in each pot. To this end, the roots were washed in running water, then the nodules were collected and dried in an oven at 65 °C for 72 h. Subsequently, they were weighed on a balance with analytical precision. The number of nodules was obtained through computerized image analysis using ImageJ software, version 1.48v [67]. The images were acquired with a Sony IMX179 sensor, with a pixel size of 1.4 μm and sensitivity of 0.65 v/lux-sec (550 nm), connected to the computer via USB 2.0 interface and positioned 12 cm from the nodules. The illumination of the samples at the time of image acquisition was standardized, using two LED panels with a power of 32 W, emission of 2200 lumens, color temperature of 6000 K, and color rendering index > 70%. Automated nodule counting was performed following the steps of Color Threshold > Analyze Particles > Summary.

The determination of BNF efficiency and N concentration was performed using the δ^15^N natural abundance method (%Ndfa) [68]. For this analysis, only the treatments of 0, 4, and 8 mL kg^−1^ of the product for seed treatment with Mo, Co, and Ni were evaluated. After collecting the aerial parts of the plants, they were dried in an oven at 65 °C for 72 h, followed by grinding to obtain material with a particle size of approximately 20–30 mesh. Samples of 5–8 mg were weighed in tin capsules and analyzed in an isotope ratio mass spectrometer, Delta V ADVANTAGE Isotope Ratio MS, Thermo Scientific. The same procedure was adopted for rice plants (non-nodulating plants), which were collected at the same time [35]. The BNF efficiency of N_2_-fixing plants, i.e., soybeans, with respective treatments, was calculated by the equation [68]:

$$\mathrm{BNF}\;=\frac{\left(\delta^{15}N\;\mathrm{reference}\;-\;\delta^{15}N\;\mathrm{soybean}\right)}{\left(\delta^{15}N\;\mathrm{reference}\;-\;B\right)}*100$$

where the following applies:

BNF—percentage of N from BNF in the soybean plant;

δ^15^N reference—natural abundance of δ^15^N in the reference plant (non-N-fixing, rice for this experiment);

δ^15^N soybean—natural abundance of δ^15^N in soybean;

B—fractionation value of δ^15^N in relation to δ^14^N by the soybean plant when absorbing N from the soil. In this research, the “B” value of −1.85 derived from the study by Guimarães et al. [69] was adopted.

### 4.5. Assessment of Plant Development

The assessments of plant development consisted of measuring the height of the plants, as well as the dry matter of the aerial part and root system. Using a tape measure, the height of the plants was determined by measuring the length of the stem, from the substrate level to the end of the central leaflet of the last trifoliate of the main stem. To determine the dry matter of the plants, the aerial parts and roots, after collection, were dried in an oven at 65 °C for 72 h. Immediately afterward, they were weighed on a precision balance.

### 4.6. Statistical Analysis

For statistical analysis, the normality of residuals and homogeneity of variances were analyzed using the Shapiro–Wilk and Bartlett tests, respectively (*p* ≤ 0.05). Data that did not meet the test assumptions were subjected to the Box and Cox transformation [70]. Then, the data were subjected to analysis of variance (ANOVA) and comparison of means using the Tukey test (*p* ≤ 0.05). Statistical analyses were performed using R software, version 4.0.5 [71], and the data were processed in SigmaPlot.

## 5. Conclusions

The seed treatment with a product containing Co, Mo, and Ni positively influenced N metabolism in soybean, particularly by enhancing urease activity, and promoted plant development through increased shoot and root dry biomass.

Although the overall trend in plant response to the applied doses was similar across seed vigor levels, certain physiological and metabolic traits—such as nitrogenase activity, nodule dry mass, and urease baseline levels—exhibited variability depending on seed vigor.

The benefits of Co + Mo + Ni application were evident regardless of seed vigor, suggesting that even plants originating from low-vigor seeds can respond favorably with Co, Mo, and Ni supplementation.

Future studies should evaluate the long-term effects of these treatments on yield components and grain quality and investigate interactions with rhizobia strains under diverse soil and climate conditions. Further elucidation of molecular mechanisms involved in the nutrient-mediated modulation of N-metabolism could also enhance precision in mineral element management strategies. In summary, this study underscores the potential of mineral element-based seed treatments as a sustainable and scalable tool for optimizing N use efficiency in soybean systems.

## Data Availability

Data are available upon request to the corresponding author.

## Appendix A

Results of the physiological potential of the seeds from lots characterized as having higher and lower vigor (HV and LV, respectively) (Table A1).

**Table A1:** Characterization of the physiological potential of two lots of soybean seeds of the M5917 IPRO cultivar, namely, lot HV and LV.

LOTE	GER	FGC	AASS	WC-AASS	EC
	%				µS cm^−1^g^−1^
HV	98	98	96	12.7	68
LV	89	83	83	12.3	102
Teste de F	*	*	^ns^	-	*
C.V. (%)	3.0	7.5	2.4	-	8.8

HV—higher vigor; LV—lower vigor; C.V.—coefficient of variation; GER—germination; FGC—first germination count; AASS—accelerated aging with saturated sodium chloride solution; WC-AASS—water content after AASS; EC—electrical conductivity; *, ns—Significant at *p* ≤ 0.05 and not significant by the F-test, respectively.
